# Measuring Discrimination in South Korea: Underestimating the Prevalence of Discriminatory Experiences among Female and Less Educated Workers?

**DOI:** 10.1371/journal.pone.0032872

**Published:** 2012-03-12

**Authors:** Seung-Sup Kim, Yeonseung Chung, S. V. Subramanian, David R. Williams

**Affiliations:** 1 Department of Environmental and Occupational Health, The George Washington University School of Public Health and Health Services, Washington, D.C., United States of America; 2 Department of Mathematical Sciences, Korea Advanced Institute of Science and Technology, Daejeon, Republic of Korea; 3 Department of Society, Human Development, and Health, Harvard School of Public Health, Boston, Massachusetts, United States of America; University of Arkansas, United States of America

## Abstract

**Objectives:**

To investigate the possibility that Koreans show different patterns in reporting discriminatory experiences based on their gender and education level, we analyzed the participants who answered “Not Applicable” for the questions of discriminatory experiences that they were eligible to answer.

**Methods:**

Discriminatory experiences in eight social situations were assessed using the 7^th^ wave of Korean Labor and Income Panel Study. After restricting the study population to waged workers, a logistic regression model was constructed to predict the probability that an individual has experienced discrimination based on the observed covariates for each of eight situations, using the data of participants who answered either Yes or No. With the model fit, the predicted logit score of discrimination (PLSD) was obtained for participants who answered Not Applicable (NA), as well as for those who answered Yes or No. The mean PLSD of the NA group was compared with those of the Yes group and the No group after stratification by gender and education level using an ANOVA model.

**Results:**

On the questions of discrimination in getting hired and receiving income, the PLSD of the NA group was significantly higher than that of the No group and was not different from that of Yes group for female and junior high or less educated workers, suggesting that their NA responses were more likely to mean that they have experienced discrimination. For male and college or more educated workers, the NA group had a PLSD similar to that for the No group and had a significantly higher PLSD than the Yes group, implying that their NA responses would mean they that they have not experienced discrimination.

**Conclusions:**

Our findings suggest that the responses of NA on the discrimination questionnaire may need different interpretation based on the respondents' gender and education level in South Korea.

## Introduction

A growing body of research is demonstrating that experiences of discrimination are associated with multiple indicators of poor health outcomes [1], [2]. Since health researchers primarily measure the experiences of discrimination through self-reports, it is important to measure discrimination accurately to examine its potential health effects. Several questionnaires with reasonable psychometric properties have been proposed to measure self-reported experiences of discrimination [3], [4]. Still, many challenges remain in measuring discrimination.

One issue of concern is the possibility that individuals may report discriminatory experiences in different ways based on their social position [5], [6], [7], [8]. That is, some individuals may deny or underreport their experiences of discrimination. Crosby (1984) suggested that there could be emotional barriers for female workers to acknowledge personal discrimination although they did not receive the rewards they deserve [9]. One experimental study in the U.S. found that, compared to men and Whites, the subordinate groups, including women, Asians and Blacks, tended to minimize their discriminatory experiences and attribute their failure to themselves because doing so was psychologically beneficial [10].

Although most studies about health effect of discriminatory experiences were conducted in US or European countries, a growing number of papers indicated that discriminatory experience is associated with high prevalence of poor health conditions in Asian countries such as Japan, Hong Kong and China [11], [12], [13]. In South Korea which has strong patriarchal tradition and pervasive educational inequality [14], [15], [16], Kim and Williams reported that gender and education level are the most common sources of self-reported discriminatory experiences and there is a dose-response relationship between the number of situation of discriminatory experiences and poor self-rated health [17].

So far, however, little attention has been given to the issue of how to accurately measure discriminatory experience in South Korea although studies in the U.S. have showed that individuals may underreport or deny discriminatory experiences based on their social positions [5], [6], . In this study, we investigate whether, in South Korea, the process of reporting discriminatory experiences is influenced by individuals' gender and educational level by analyzing the participants who answered “Not Applicable” for the questions of discriminatory experiences that they were eligible to answer.

## Methods

### Data Description

Data were obtained from the 7^th^ wave of *The Korean Labor and Income Panel Study* (KLIPS), an annual longitudinal in-person survey of labor and employment for a representative sample of Korean households and individuals launched in 1998 [http://www.kli.re.kr/klips]. The KLIPS recruited about 5,000 households in urban areas using two-stage stratified cluster sampling at baseline and the data from the 1^st^ to the 11^th^ waves (1998–2008) have been released to the public. Our analyses only included the survey participants who were employed at the time of the 7^th^ wave of survey (2004) in order to restrict the study population to those who were eligible to answer the questions on work-related discriminatory experiences.

### Measurement of perceived discriminatory experience

The discrimination questionnaire was administered at the 7^th^ wave of the survey (2004) by trained personnel. Discriminatory experiences were measured using a modified version of the “Experience of Discrimination” (EOD) questionnaire which asked participants whether they have “ever experienced discrimination” in each of eight situations: in getting hired, in receiving income, in training, in getting promoted, in being fired, in obtaining higher education, at home, and in general social activities [4]. The first five situations were about work-related discriminatory experiences and the other three situations were about discriminatory experiences outside workplaces. For each question, participants could answer Yes, No, or Not Applicable (NA). Although we expected that all participants in the study population were eligible to answer these questions because all of them were waged workers at the time of survey, a large number of respondents answered NA (Table 1).

**Table 1:** Distribution of the discriminatory experiences in eight different situations for the waged workers at the 7^th^ wave of the Korean Labor and Income Panel Study in South Korea (N = 3,594).

Situations of discriminatory experiences	Survey participants' responses		
	Yes	No	Not Applicable
	N (%)	N (%)	N (%)
Hired	680 (18.9)	2,762 (76.9)	152 (4.2)
Income	541 (15.1)	2,944 (81.9)	109 (3.0)
Training	71 (2.0)	3,101 (86.3)	422 (11.7)
Promotion	209 (5.8)	2,893 (80.5)	492 (13.7)
Fired	64 (1.8)	3,064 (85.3)	466 (13.0)
Education	38 (1.1)	3,343 (93.0)	213 (5.9)
Home	73 (2.0)	3,431 (95.5)	90 (2.5)
Social activities	280 (7.8)	3,233 (90.0)	81 (2.3)

### Covariates in prediction model

**Figure 1:**
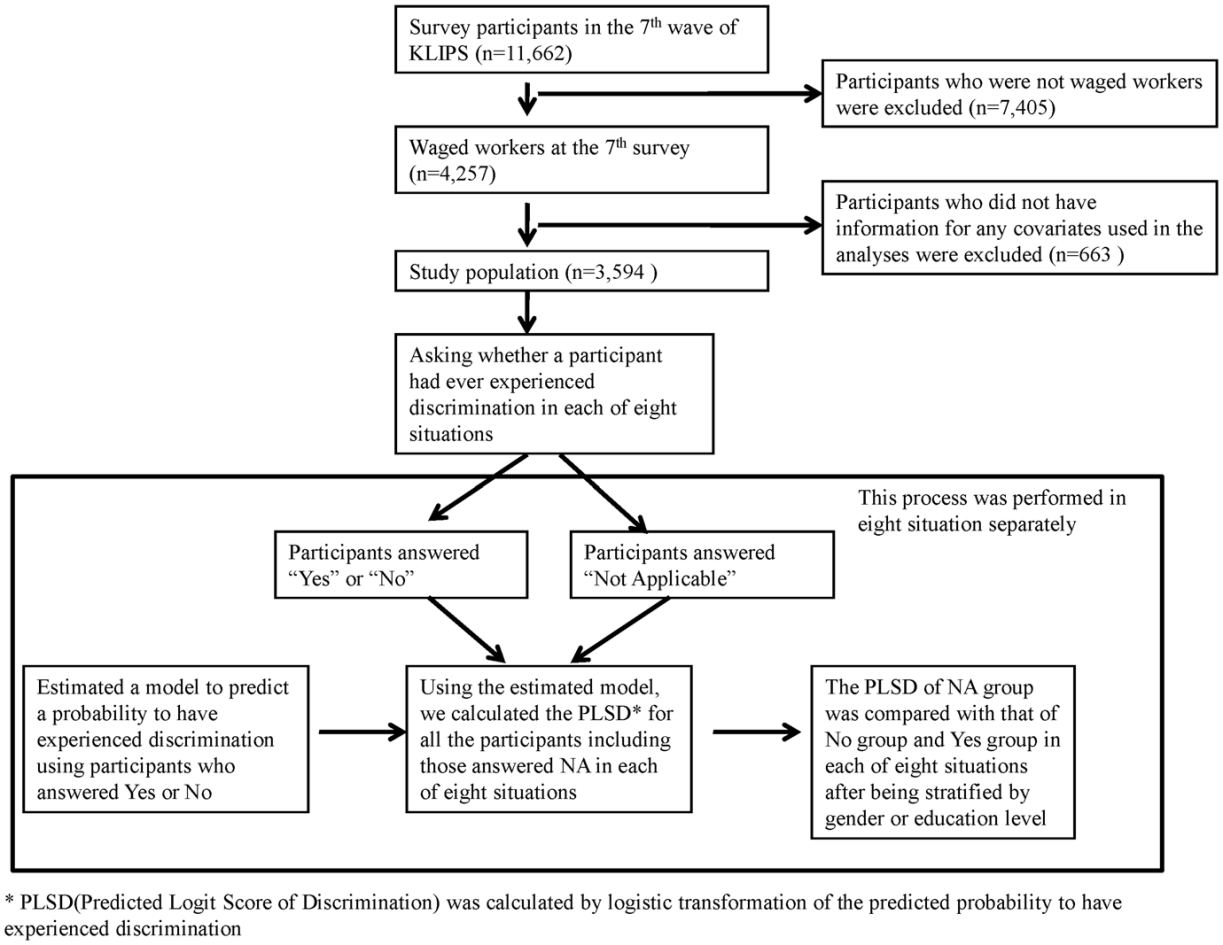
Flow chart of data analyses. * PLSD (Predicted Logit Score of Discrimination) was calculated by logistic transformation of the predicted probability to have experienced discrimination based on workers' socio-demographic and health-related variables in each of eight situations.

To build prediction models for the probability that an individual have experienced discrimination, the observed covariates were used as predictors in the logistic regressions. The covariates used in the model were obtained from the 7^th^ wave of the survey including gender, age, education level (junior high or less, high school graduate, college graduate or more), marital status (never, currently, previously), employment status (precarious, non-precarious), household equivalent income, birth region, and self-rated health condition. In addition, individual disability information was obtained from the 9^th^ wave of the survey because the disability data were collected only at that survey time. The distributions of all the covariates are shown in Table 2. Individuals with missing values in any of the covariates were excluded from the study (Figure 1).

**Table 2:** Distribution of the study population and prevalence of discriminatory experience stratified by covariates among the waged workers at the 7^th^ wave of the Korean Labor and Income Panel Study (2004) in South Korea (N = 3,594).

	Distribution	Prevalence of any discriminatory experiences	
	N (%)	N (%)	P-value*
Gender			
Male	2,174 (60.5)	541 (24.9)	
Female	1,420 (39.5)	431 (30.4)	<0.001
Age (years)			
16–25	271 (7.5)	80 (29.5)	
25–34	1,132 (31.5)	261 (23.1)	
35–44	1,038 (28.9)	250 (24.1)	
45–54	748 (20.8)	225 (30.1)	
55–64	306 (8.5)	110 (36.0)	<0.001
65+	99 (2.8)	46 (46.5)	
Education			
Junior high or less	736 (20.5)	308 (41.9)	
High school graduate	1,379 (38.4)	398 (28.9)	
College graduate or more	1,479 (41.2)	266 (18.0)	<0.001
Marital status			
Previously married	233 (6.5)	93 (39.9)	
Never married	877 (24.4)	241 (27.5)	
Currently married	2,484 (69.1)	638 (25.7)	<0.001
Employment status			
Precarious employment	840 (23.4)	320 (38.1)	
Non-precarious employment	2,754 (76.6)	652 (23.7)	<0.001
Household income			
Less than 1Q	898 (25.0)	332 (37.0)	
1Q-2Q	898 (25.0)	276 (30.7)	
2Q-3Q	898 (25.0)	199 (22.2)	
3Q-	900 (25.0)	165 (18.3)	
Having a disability			
No	3,501 (97.4)	930 (26.6)	
Yes	93 (2.6)	42 (45.2)	<0.001
Birth region			
Cholla province	745 (20.7)	193 (25.9)	
Other regions	2,849 (79.3)	779 (27.3)	0.432
Self-rated health condition			
Very Good	168 (4.7)	36 (21.4)	
Good	2,056(57.2)	536 (26.1)	
Fair	1,133 (31.5)	304 (26.8)	
Poor	226 (6.3)	89 (39.4)	
Very Poor	11 (0.3)	7 (63.6)	<0.001

* P-value of the Chi-square test comparing the prevalence of any discriminatory experiences across different categories of each covariate.

Employment status was divided into two categories: precarious and non-precarious employees. Such categorization was motivated by the studies showing that precariously employed workers are more disadvantaged compared to non-precarious ones in terms of wages, labor union support, social benefits, and health-related conditions [18], [19]. Precarious employment includes temporary employment, daily employment, or part-time employment. All workers who were not classified as precarious workers were categorized as non-precarious workers. Household equivalent income was calculated by dividing the total household income by the square root of the number of household members. We created four income categories based on the quartiles of the results of that formula. Birth region was separated into Cholla province versus other regions, because individuals born in Cholla province have been politically and economically isolated in South Korea and historically stigmatized [20], [21]. Disability was measured only at the 9^th^ wave of data collection and was assessed by the question “Do you have any impairment or disability?”. Self-rated health condition was originally measured with a five-point scale from ‘excellent’ (score 1) to ‘very poor’ (score 5) for the question, “How would you rate your health?”. Because of the skewed distribution, in all of our analyses the two categories of self-rated health (very poor and poor) were merged into one.

### Data Analyses

Preliminarily, the prevalence of perceived discriminatory experience was compared across different socio-demographic groups using the Chi-square test (p-values are reported in Table 2). To investigate the participants who answered “Not Applicable” for the questions of discriminatory experiences that they were eligible to answer, our analysis proceeded as follows. First, a logistic regression was used to build prediction models for the probability (p) of having experienced discrimination in each of eight situations (equivalently, the logit score of discrimination (log(p/(1-p))) based on the observed covariates including socio-demographic and health-related variables. The models were trained using the data for the participants who answered either Yes or No. Age was included as a continuous covariate in the model, and all others were considered as categorical covariates. We used age as a linear term because any other higher-order terms were not statistically significant (p-values >0.05). The model formula is written as:
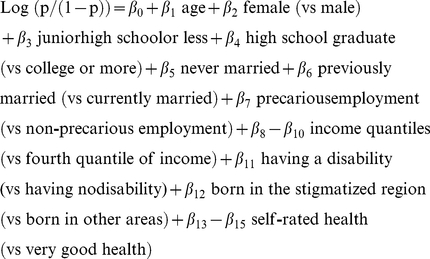



Based on the above model fit, we obtained the predicted logit score of discrimination (PLSD) for participants who answered NA, as well as for those who answered Yes or No. We confirmed that the range of covariate values for the NA group was covered by the Yes or No group, thus our prediction for the NA group is reasonable. The mean PLSD of the NA group was then compared with the Yes group and with the No group using an ANOVA model after stratification by gender or by education level (junior high or less educated, high school graduate, college or more educated). The model was parameterized as mean (PLSD) = α_0_+α_1_ 1(Yes)+α_2_ 1(No) so that α_1_ and α_2_ represent the differences in the mean PLSD between the NA and Yes groups and between the NA and No groups, respectively. Gaussian error for the PLSD was assumed and checked as appropriate. Two-sided p-values were presented in the tables. All analyses were performed using STATA/SE version 11.0 (StataCorp, College Station, TX).

### Ethics

The KLIPS is the publicly released dataset that is available at the website of Korea Labor Institute (http://www.kli.re.kr/). Informed consent was not required to use this dataset. This research received IRB exemption from the Office of Human Research Administration at the Harvard School of Public Health.

## Results

Table 2 summarizes the distribution of the study population across each covariate and the prevalence of any lifetime discriminatory experiences (if experienced in at least one of the eight situations) stratified by covariates. Significantly higher prevalence of any lifetime discriminatory experiences were observed for participants who were female, older, less educated, previously married, precariously employed, with lower household income, and disabled than their counterparts. The prevalence of any lifetime discriminatory experience did not vary depending on birth region.

After fitting the prediction model, we obtained the PLSD for all participants and compared the mean PLSD among the three different response groups (Yes, No, NA) for each of eight situations within each education level and each gender (Tables 3 and 4). In general, the PLSDs of the NA group fell between those of the Yes group and No group in all situations except in getting promoted, at home and at general social activities, where some of the NA group had a lower PLSD than both the Yes group and No group. Further, in two situations of discriminatory experiences (i.e. getting hired and receiving income), there was a consistent trend regardless of stratification. The PLSD of the NA group was significantly higher than that of the No group but was not different from that of the Yes group for female or junior high or less educated workers. For male and college or more educated workers, the NA group had a similar PLSD to the No group but a significantly lower PLSD than the Yes group.

**Table 3:** Comparison of the mean PLSD^a^ among three different response groups of the waged workers at the 7^th^ wave of The Korean Labor and Income Panel Study (2004) within each education level in South Korea (N = 3,594).

Situations of discriminatory experiences	Responses	Junior high or less (N = 736)			High school (N = 1,379)			College or more (N = 1,479)		
		α	95% CI		α	95% CI		α	95% CI	
Hired	Not Applicable	0	Referent		0	Referent		0	Referent	
	Yes	0.01	−0.18	0.19	0.19**	0.06	0.32	0.35***	0.17	0.53
	No	−0.21*	−0.39	−0.03	−0.03	−0.15	0.09	−0.07	−0.22	0.09
Income	Not Applicable	0	Referent		0	Referent		0	Referent	
	Yes	−0.05	−0.22	0.13	0.04	−0.08	0.17	0.29**	0.08	0.49
	No	−0.19*	−0.36	−0.02	−0.10	−0.21	0.02	−0.13	−0.31	0.05
Training	Not Applicable	0	Referent		0	Referent		0	Referent	
	Yes	0.54***	0.25	0.83	0.22	−0.01	0.45	0.24	−0.03	0.51
	No	0.01	−0.11	0.13	−0.03	−0.12	0.07	−0.11	−0.22	0.01
Promotion	Not Applicable	0	Referent		0	Referent		0	Referent	
	Yes	0.53***	0.34	0.71	0.25***	0.15	0.36	0.13*	0.01	0.24
	No	0.10*	0.02	0.19	0.1**	0.03	0.16	0.04	−0.04	0.11
Fired	Not Applicable	0	Referent		0	Referent		0	Referent	
	Yes	0.25	−0.06	0.55	0.38**	0.13	0.62	0.38*	0.00	0.77
	No	−0.32***	−0.47	−0.17	0.01	−0.09	0.11	−0.13**	−0.23	−0.04
Education	Not Applicable	0	Referent		0	Referent		0	Referent	
	Yes	1.29***	0.76	1.82	0.72	−0.13	1.58	1.05	−0.15	2.25
	No	−0.25	−0.53	0.02	−0.14	−0.39	0.12	−0.26	−0.65	0.12
Home	Not Applicable	0	Referent		0	Referent		0	Referent	
	Yes	1.17	−0.06	2.39	3.13***	1.48	4.78	3.03**	1.09	4.97
	No	−0.27	−1.32	0.78	1.13*	0.12	2.15	0.53	−0.55	1.61
Social activities	Not Applicable	0	Referent		0	Referent		0	Referent	
	Yes	−0.12	−0.36	0.12	0.11	−0.03	0.25	0.09	−0.06	0.24
	No	−0.31**	−0.53	−0.08	−0.04	−0.17	0.09	0.00	−0.13	0.12

* p<0.05,

** p<0.01,

*** p<0.001.

a PLSD (Predicted Logit Score of Discrimination) was calculated by logistic transformation of the predicted probability to have experienced discrimination based on workers' socio-demographic and health-related variables in each of eight situations.

**Table 4:** Comparison of the mean PLSD^a^ among three different response groups of the waged workers at the 7^th^ wave of The Korean Labor and Income Panel Study (2004) within each gender in South Korea (N = 3,594).

Situations of discriminatory experiences	Responses	Females (N = 1,420)			Males (N = 2,174)		
		α	95% CI		α	95% CI	
Hired	Not Applicable	0	Referent		0	Referent	
	Yes	0.01	−0.18	0.20	0.68***	0.51	0.85
	No	−0.37***	−0.55	−0.19	0.07	−0.08	0.23
Income	Not Applicable	0	Referent		0	Referent	
	Yes	−0.04	−0.24	0.17	0.66***	0.44	0.87
	No	−0.36***	−0.55	−0.17	0.07	−0.13	0.27
Training	Not Applicable	0	Referent		0	Referent	
	Yes	0.19	−0.06	0.44	0.35**	0.12	0.59
	No	−0.10*	−0.19	−0.01	−0.27***	−0.37	−0.16
Promotion	Not Applicable	0	Referent		0	Referent	
	Yes	0.23***	0.11	0.36	0.21***	0.12	0.30
	No	0.11**	0.05	0.18	−0.01	−0.07	0.05
Fired	Not Applicable	0	Referent		0	Referent	
	Yes	0.99***	0.61	1.36	0.51***	0.23	0.80
	No	−0.14**	−0.27	−0.01	−0.09	−0.20	0.03
Education	Not Applicable	0	Referent		0	Referent	
	Yes	1.13***	0.64	1.62	0.55	−0.40	1.50
	No	−0.48***	−0.72	−0.23	−0.41**	−0.65	−0.18
Home	Not Applicable	0	Referent		0	Referent	
	Yes	1.63**	0.59	2.67	2.59*	0.44	4.75
	No	0.60	−0.23	1.43	1.12**	0.27	1.96
Social activities	Not Applicable	0	Referent		0	Referent	
	Yes	0.05	−0.14	0.25	0.55***	0.36	0.75
	No	−0.11	−0.28	0.06	0.08	−0.09	0.26

* p<0.05,

** p<0.01,

*** p<0.001.

a PLSD (Predicted Logit Score of Discrimination) was calculated by logistic transformation of the predicted probability to have experienced discrimination based on workers' socio-demographic and health-related variables in each of eight situations.

Within each education level, the PLSD of the NA group was compared with the Yes and No groups (Table 3). For junior high or less educated workers, the PLSD of the NA group was similar to that of the Yes group and significantly higher than that of the No group in four situations: getting hired, receiving income, getting fired, and at social activities. However, in training and obtaining higher education, the NA group was similar in PLSD to the No group and significantly higher than the Yes group. For high school or more educated workers, the PLSD of the NA group did not differ from either that of the Yes group or the No group in experiencing discrimination in getting training, obtaining higher education, and at social activities.

Within each gender, the NA group had a significantly higher PLSD than the No group in all situations except for at home and at social activities for females (Table 4). Additionally, the female NA group was similar in PLSD to the Yes group in getting hired, receiving income, getting training, and at social activities. In contrast, the male NA group was similar in PLSD to the male No group in five situations: getting hired, receiving income, getting promoted, getting fired, at home, and at social activities.

## Discussion

The results of this paper suggest that the response of NA on the discrimination questionnaire needs different interpretation based on participants' gender and education level in South Korea. Our results showed, when female or less-educated workers answered NA for the questions of discriminatory experiences in getting hired and in receiving income despite their eligibility, they were similar to people who have experienced discrimination, implying that their NA responses were more likely to mean that they have experienced discrimination. In contrast, when male and college or more educated workers responded NA to the same questions, they were more likely to mean that they have not experienced discrimination.

These findings are consistent with previous studies in the US showing that subordinate groups like women or African-Americans are more likely to deny or under-report discriminatory experiences compared to dominant groups like men and whites [6], [9], [22]. In addition, research about personal/group discrimination discrepancy also shows that people in “disadvantaged” groups, such as African-American women and Asian female immigrants, in the US and Canada tend to report less discrimination for themselves than for their group, implying under-reporting of personal experiences of discrimination [23], [24], [25].

Various explanations describe this differential reporting of discriminatory experiences. Several factors, including conscious or unconscious denial, positive coping, optimism, and internalized oppression, have been suggested to be influential on an individual's reporting of discriminatory experience [5]. For example, our findings may result from internalized oppression in female and junior high or less educated workers. Those subordinate groups are more likely to perceive unfair treatment as deserved, because they internalize negative attitudes toward themselves by accepting the dominant culture's definition of their role or status in society [6], [7], [8]. Thus, these individuals might answer NA although they actually have experienced discrimination. Another potential mechanism is that participants might have shaped their answer to be “socially acceptable.” Since the survey was conducted by trained interviewers, interviewer bias may have influenced the respondents' answers due to the sensitive nature of the topic of discrimination [26].

We assumed that all participants in the study population were eligible for the questionnaire of discriminatory experiences because they were waged workers at the time of study. Eligibility was clear for the questions regarding discriminatory experiences in getting hired, receiving income, obtaining higher education, at home and at general social activities, but not for the other work-related situations of getting training, promoted, and fired. For example, precarious workers such as day laborers, temporary workers, and part-time workers, may not have been exposed to the chance of getting training, promoted, or fired because of the nature of their jobs. Therefore, respondents who answered NA in getting training, promoted, and fired may represent a group of people who were not actually eligible for the question. This explanation is plausible because a higher proportion of participants reported NA in those three work-related situations compared to other situations (Table 1). Future studies need to address this issue by using people's detailed employment history or asking people's discriminatory experience in current workplace during last year.

This study indicates the potential for biased estimation of reported experiences of personal discrimination in South Korea. For examples, there could be several scenarios which could lead to biased estimation of discriminatory experiences in dealing with the survey participants who responded NA for the questions of discriminatory experiences in getting hired and receiving income. First, if a questionnaire of discriminatory experiences provides only two available answers, either Yes or No, but not NA, then the NA group might answer No. Second, a researcher may treat NA as No in the data analysis, assuming that the NA group did not experience discrimination. Third, the prevalence of reported experiences of discrimination may be calculated after excluding the NA group. If our findings are replicated, using these approaches are likely to result in underestimating the prevalence of discriminatory experiences in the female and junior high or less educated workers (all the three scenarios) and overestimating the prevalence in the male and college or more educated workers (the third scenario). Thus, a differential misclassification of discriminatory experience would result, which could lead to the biased estimation of health effects from discriminatory experiences. The extent to which, a similar pattern may exist in other contexts needs to be explored. Our observation offers suggestive evidence for why a “J-shaped” relationship has been observed between racial discrimination and poor health with under-reporting of discrimination in the subordinate group [5], [7].

The major limitation of this study is that we have no data on the validity of the “Experience of Discrimination” questionnaire for South Korea. The questionnaire was developed to assess the degree of racial or gender discrimination in the US and has been validly and reliably employed with working-class African- and Latino-Americans [4], [27]. However, measurement of discriminatory experience can be sensitive to cultural differences and contexts, so further study is required to assess the validity and reliability of this instrument in South Korea.

To our knowledge, this is the first study to show different patterns of reporting discriminatory experiences based on gender and education level in South Korea which has strong patriarchal tradition and pervasive educational inequality [14], [15], [16]. This study suggests that the response of NA on the discrimination questionnaire may need different interpretation based on the respondents' gender and education level in South Korea. On the questions of discriminatory experiences in getting hired and receiving income, the PLSD of the NA group was significantly higher than that of the No group and was not different from that of Yes group for female and junior high or less educated workers, suggesting that their NA responses are more likely to mean that they have experienced discrimination. For male and college or more educated workers, the NA group had a PLSD similar to that for the No group and had a significantly higher PLSD than the Yes group, implying that their NA responses would mean they that they have not experienced discrimination. This phenomenon could lead to potential underestimation of discriminatory experience in the female and junior high or less educated workers.
